# Monitoring and analysis of dynamic growth of human embryonic stem cells: comparison of automated instrumentation and conventional culturing methods

**DOI:** 10.1186/1475-925X-6-11

**Published:** 2007-04-12

**Authors:** Susanna Narkilahti, Kristiina Rajala, Harri Pihlajamäki, Riitta Suuronen, Outi Hovatta, Heli Skottman

**Affiliations:** 1 REGEA, Institute for Regenerative Medicine, University of Tampere and Tampere University Hospital, 33520 Tampere, Finland; 2 The Finnish Defence Forces, 00131 Helsinki, Finland; 3 Karolinska Institute, CLINTEC, Karolinska University Hospital Huddinge, 14186 Stockholm, Sweden

## Abstract

**Background:**

Human embryonic stem cells (hESCs) are a potential source of cells for use in regenerative medicine. Automation of culturing, monitoring and analysis is crucial for fast and reliable optimization of hESC culturing methods. Continuous monitoring of living cell cultures can reveal more information and is faster than using laborious traditional methods such as microscopic evaluation, immunohistochemistry and flow cytometry.

**Methods:**

We analyzed the growth dynamics of two hESC lines HS237 and HS293 in a conventional culture medium containing serum replacement and a xeno-free X-vivo 10 medium. We used a new automated culture platform utilizing machine vision technology, which enables automatic observation, recording and analysis of intact living cells. We validated the results using flow cytometry for cell counting and characterization.

**Results:**

In our analyses, hESC colony growth could be continuously monitored and the proportion of undifferentiated cells automatically analyzed. No labeling was needed and we could, for the first time, perform detailed follow up of live, undisturbed cell colonies, and record all the events in the culture. The growth rate of the hESCs cultured in X-vivo 10 medium was significantly lower and a larger proportion of the cells were differentiated.

**Conclusion:**

The new automated system enables rapid and reliable analysis of undifferentiated growth dynamics of hESCs. We demonstrate the effectiveness of the system by comparing hESC growth in different culture conditions.

## Background

Traditionally, cell cultures are monitored by time consuming microscopy, manual imaging and image processing. Conventional time-lapse recordings have provided new data of living cell behavior and growth. Despite the advantages, the technique has drawbacks in particular the length of follow-up time. In addition, monitored areas of interest are limited, and analysis of data is not automated. We tested a new technology platform for continuous monitoring and analysis of living cell cultures. The system applies machine vision technology to analyze and quantify morphologic traits. Events such as apoptosis, cell division, cellular movement, attachment, and the number of single cells can be continuously observed and recorded for up to several weeks, if needed. The optical design utilizes a dynamic z-stack to produce all-in-focus [1-3] information rich images enabling detailed analysis by generating in-depth images not previously seen with other techniques. Machine vision technology has traditionally been employed in the fields of medical imaging, precision robotics, and on factory assembly lines for consistently differentiating shape, size, position, patterns and movements [4,5].

Human embryonic stem cells (hESCs) are pluripotent cells capable of self-renewal and differentiation into all cell types in the body [6,7]. They hold great potential for regenerative medicine. Significant quantities of hESCs are needed for differentiation to a final phenotype for cell transplantation. Current culture methods are not capable of producing adequate quantities of hESCs at an acceptable quality and price for cell transplantation. Improved automated culture analyses for undifferentiated hESCs are desired. In this report we describe, for the first time, the growth dynamics of hESCs using a new automated culture and monitoring system. Using this system, we compare conventional culture medium (containing animal protein serum replacement) to a xeno-free culture medium.

## Methods

### Human ESC cultures

Human ESC lines HS237 (passages 63, 70, and 80) and HS293 (passages 58, 64, and 78), derived at Karolinska University Hospital Huddinge, Karolinska Institutet, Sweden, were cultured as previously described [8,9]. The hESC colonies were mechanically divided and seeded as small aggregates onto 12-well plates (CellBIND Surface, Corning, Inc., Corning, NY), containing gamma-irradiated human foreskin fibroblasts (CRL-2429, ATCC, Manassas, VA) as feeder cells.

The hESCs were cultured either in a conventional medium or in an X-vivo 10 medium. The conventional hESC medium contained 80% (vol/vol) KnockOut Dulbecco's Modified Eagle's Medium (DMEM) and 20% (vol/vol) KnockOut serum replacement supplemented with 2 mM Glutamax, 0.1 mM β-mercaptoethanol, 0.1 mM MEM non-essential amino acids (Cambrex Bio Science, Walkersville, Inc., Walkersville, MD), 50 U/ml penicillin-50 μg/ml streptomycin (Cambrex Bio Science) and 8 ng/ml recombinant human fibroblast growth factor (bFGF, R&D Systems, Minneapolis, MN). The X-vivo 10 medium contained X-VIVO 10 (Cambrex Bio Science) medium, 0.12 ng/ml transforming growth factor β1 (Sigma, St. Louis, MO) and the same supplements included in the conventional hESC medium. All reagents were from Invitrogen (Invitrogen, Carlsbad, CA) unless stated otherwise. Before analysis, the hESCs were slowly adapted to the X-vivo 10 medium by gradually increasing the proportion of X-vivo 10 medium and decreasing the proportion of conventional hESC medium.

### Instrumentation

**Figure 1:**
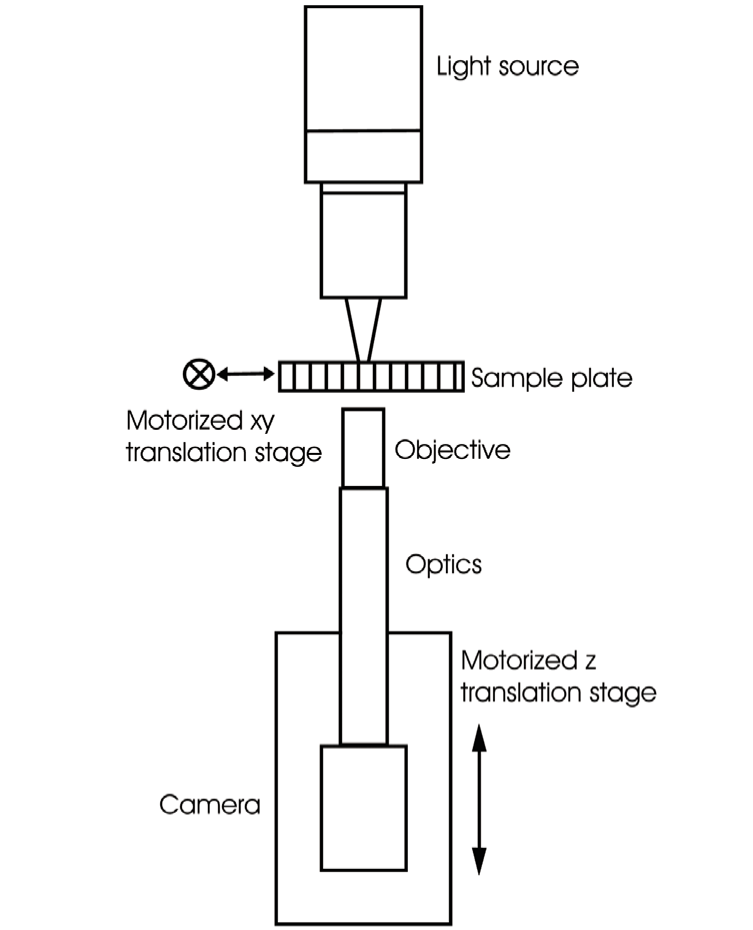
A schematic drawing of the automated optics module and sample monitoring system. The automated optics module consists of a digital 768 × 576 pixel CCD Camera (Jai cv-A10CL) coupled to a phase-contrast microscope with a 10× phase-contrast objective (Nikon CFI Achromat) and 200 mm optics (Infinity Infinitube), producing a 675 × 506 μm field – of – view with a spatial resolution of 0.879 μm/pixel, built onto a motorized z-direction movement stage. The illumination source is a green (530 nm) LED light that enables high quality phase-contrast images. More information [15].

The Cell-IQ^® ^system (Chip-Man Technologies Ltd., Tampere, Finland) is a self – contained cell culturing instrument combining phase-contrast microscopy, environmental control, and automation (Table 1). The instrument contains an automated optics module (Figure 1), an integrated incubator (+/- 0.2°C), 2 incubation gas flow controllers, precision movement stages (x, y axes: ± 1 μm; z axis: ± 0.4 μm) fully controlled through machine vision – based firmware and analysis software. The imaging system enables continuous monitoring of adherent cells in two plates in an integrated plate holder. Through pattern recognition, individual cells can be automatically located and monitored after culture media changes. Machine vision enables analysis of a continuous time-lapse image series of living cells for observing morphologic changes without the use of labels and dyes.

**Table 1:** The Cell-IQ^® ^instrument compositions

**CELL-IQ MACHINE VISION SYSTEM**		
**Phase contrast microscope**	**Environment control**	**Automation**

• Light source	• Temperature control and logging	• Automated cell imaging for selected positions
• Optics	• Incubation gas control	• Label free cell analysis based on morphology
• Digital camera		• Motorized XYZ translation stages

#### Perfusion lid

The incubation gases are piped directly onto the culture plate through a perfusion lid. The lid is designed with inlet and outlet connectors. The connectors are positioned so that gas can be piped under a purified stop-flow regime to enable optimal well concentrations of the incubation gas across the plate. The gas mixture is user-defined and is under automatic control of the instrument. Two different gases can be piped onto a single plate to allow, for example, hypoxia studies.

#### Dynamic Z-stack

To enable the generation of all-in-focus images (675 × 506 μm) of irregularly spaced objects, the detector unit has an automatic z-focus control utilizing a dynamic z-stack (user-defined) that translates all objects within the z-stack into a single planar focused image. The best focus position can change during the test. The system compensates the z-position changes by adjusting the focus automatically each time the system uses the z-stack information.

#### Grid View

To monitor larger areas or objects like cell colonies, a larger field-of-view (FOV) can be generated. In these FOVs, 1 × 1 to 12 × 12 positions can be combined into a stitched grid view automatically. The monitored positions in these grids are stitched together as one image to enable easy review and analysis.

#### Autofocus

The system has an autofocus based on the detection of high frequencies from digital images. Autofocus is useful when monitoring adherent monolayer cell populations, as the well plate surfaces are uneven and the same focus cannot be used for all positions. When the target is tens of micrometers thick, such as in hESC colonies, there is a large z-range of equally good focus, and hence the autofocus is not recommended. In these situations the user sets the focus manually for each position – or copies the same focus for several positions.

#### Machine vision

Machine vision technology uses an imaging system and computer for analysis and makes decisions based on that analysis [10]. The Cell-IQ system utilizes machine vision approaches that allow monitoring and analysis of objects such as cell cultures without indicator labels. In the system an Information-Rich-Image (IRI) is generated. This IRI contains all the necessary information for an automatic analysis without human intervention. The system finds all objects in these images. It then extracts several features from these objects and uses them for classification. The classification module is based on statistical classifiers, which can be 'taught' to determine morphologic changes occurring within the culture even at the single cell level.

#### The hESC area measurement analysis method

We developed a new hESC area analysis method. The system recognizes every pixel in each image and assigns the pixel to one of the user defined classes. The colony area measurement classes were 1) background feeder layer, 2) undifferentiated area, and 3) differentiated area [see Additional file 1]. The total analyzed area in one image is normally 360448 pixels = (768-(2*32)) × (576-(2*32). Where 768 is the image width and 576 is the image height. The 32 pixel-wide strip from the image edges is not analyzed due to method requirements. The area analysis method results in total square pixel number for each of the classes in every time-lapse image and that number can be converted into the μm data. The area protocol designed here does not count single cells or the growth of the single cells in compact hESC colonies grown on top of the feeder layer. Briefly, for the analysis method development [see Additional file 2], colonies were first visually evaluated by experienced observer using conventional microscope. Colonies were photographed and undifferentiated and differentiated areas were marked on the images. Next, colonies were imaged with Cell-IQ system. Captured images were viewed in Cell-IQ Analyser program and Pick Samples Mode was used to collect representative area samples (64 × 64 pixel) for each three classes described above [see Additional file 1]. Area samples were collected from the colonies according to the microscopic evaluation. For each class, ~100 samples were collected. Thereafter, sample file was send to the company with Cell-IQ images of the colonies where the analysis protocol was built and tested for correct area recognition. Next, we tested the designed area protocol for image series, and made few corrections to the sample file in order to improve the correct recognition.

### Human ESC monitoring

On days 1 through 3 after passaging of hESCs, the plates were transferred to the monitoring equipment. Using the instrument's imaging software, the images from selected plate positions were recorded as grid images and stored in separate folders in JPEG format [11]. Every region of interest was monitored every 40 to 60 minutes. Colonies were monitored until day seven after passaging.

### Validation of automated analyses

#### Growth of hESC colony area

To cross-validate the growth data of the colonies, all colonies were photographed using a Nikon Eclipse TE2000-S phase-contrast microscope, a Nikon DS-5M camera and Eclipse Net software (version 1.20), enabling manual hESC colony area measurements.

#### Immunohistochemistry

The colonies were fixed in culture dishes with 4% paraformaldehyde in phosphate-buffered saline (0.01 M PBS, pH 7.4) for 20 min at room temperature (RT) followed by washing with PBS (2 × 5 min). The cells were permeabilized and blocked with 0.1% Triton X-100, 1% bovine serum albumin (BSA, Sigma), and 10% normal donkey serum (Sigma) in PBS for 45 min at RT and washed once with 0.1% Triton X-100, 1% BSA, and 1% normal donkey serum in PBS. The cells were incubated with the primary antibodies, polyclonal goat anti-human Nanog at a dilution of 1:200 and monoclonal mouse anti-human SSEA-1 at 1:200 (both from Santa Cruz Biotechnology, Inc., Santa Cruz, CA), overnight at 4°C. Cells were washed (3 × 5 min) with 1% BSA in PBS and probed with the secondary antibodies, rhodamine red-conjugated donkey anti-mouse IgM at 1:400 (Jackson ImmunoResearch Laboratories, West Grove, PA) and Alexa Fluor 488 donkey anti-goat IgG at 1:800 (Molecular Probes, Carlsbad, CA), for 1 h in the dark at RT. After incubation, the cells were washed with PBS (3 × 5 min) and mounted using Vectashield mounting medium containing DAPI (Vector Laboratories, Inc., Burlingame, CA). Labeled cells were viewed and photographed with a Nikon Eclipse TE2000-S phase-contrast microscope with fluorescence optics and a Nikon COOLPIX 5400 camera.

#### FACS analysis

A total of 143 colonies of the HS237 line cultured in the Cell-IQ equipment was used in fluorescence-activated cell sorting (FACS) analysis. The cells were enzymatically removed from the culture dish with Tryple™ Select (Invitrogen) for 15 min at 37°C, resuspended in 1 ml FACS buffer I (2% FBS, 0.01 % sodium azide in PBS), and counted with Neubauer cell counter chambers. Total of 3 million cells were collected and divided into samples containing 100 000 cells. The cells were probed for 15 min at 4°C with a 1:500 dilution of monoclonal mouse anti-human SSEA-4 (Santa Cruz Biotechnology, Inc.) in FACS buffer I. They were then washed in FACS buffer I and probed in FACS buffer I containing a 1:500 dilution of r-phycoerythrin-conjugated goat anti-mouse IgG (Caltag Laboratories, Carlsbad, CA) or an appropriate isotype control for 15 min in the dark at 4°C. The cells were then washed once with FACS buffer I, once with FACS buffer II (0.01% sodium azide in PBS), and fixed with 1% formaldehyde in PBS. The samples were analyzed using FACSAria™ equipment (BD Biosciences, Franklin Lakes, NJ). The cell population of interest was determined and dead cells excluded using forward and side scatter parameters. Acquisition was set for 10 000 events per sample. The data was analyzed with FACSDiva Software (version 4.1.2). Triplicate samples were analyzed in each experiment.

### Statistics

All statistical analyses were performed with SPSS for Windows software (V9.0) using a nonparametric test (the Kruskall-Wallis test) followed by *post hoc *analysis (the Mann-Whitney *U*-test). A *P*-value of < 0.05 was considered significant. Differences between the hESC lines and culture conditions were tested using Wilcoxon's signed rank test.

## Results and discussion

We applied and further developed the new automated monitoring and analysis platform with the goal of comparing hESC cultures in two different culture media. The platform enabled us to monitor growing hESC colonies. A grid-format picture capture system allowed the monitoring of living hESC cultures ranging in size from a few hundred micrometers to cell colonies of several millimetres at a defined picture capture cycle rate. The captured images were visualized with analysis software as movies, which were further used for the analysis of the colony growth.

**Figure 2:**
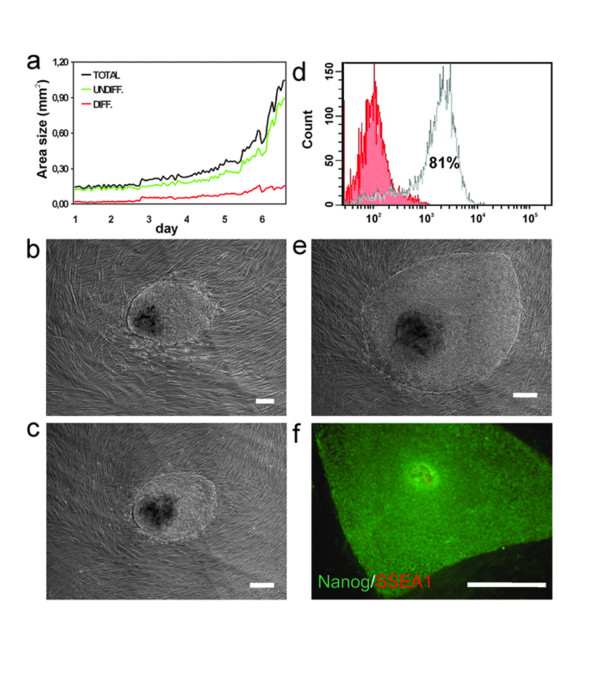
**Characterization of hESC colonies cultured in conventional hESC medium**: (**a**) A representative growth graph of an HS293 colony cultured using the automated culture platform for 7 d after passaging. The growth rate of the colony was most rapid from day 5 onwards. An HS293 colony at day 1 **(b)**, day 3 **(c)**, and day 7 **(e) **after passaging, monitored using the automated system. (**d**) FACS analysis of HS237 colonies at day 7 after passaging. The red column represents the negative control sample and the white column represents SSEA4-positive cells (81%). An HS293 colony monitored with the automated system was immunohistochemically characterized **(f) **using an antibody against an undifferentiated hESC marker, Nanog (green), and a differentiated hESC marker, SSEA1 (red). Scale bar 200 μm (**b,c,e**), 100 μm (**f**).

The hESCs were cultured in a defined atmosphere (5% CO_2 _and 36.5°C) from days 1 to 7 after passaging (Figure 2a). The hESC colonies attached to the feeders on days 1 to 2 after passaging (Figure 2b), and outgrowth of the hESC colonies started at day 3 (Figure 2c). At day 7, the hESC colonies had reached a size where they needed to be passaged (Figure 2e). Using the automated system, continuous monitoring of hESC colony growth was feasible [see Additional file 4]. We analyzed the total area and the areas of differentiated and undifferentiated cells in the hESC colonies at day 6 after passaging [see Additional file 3]. Totally differentiated and non-growing hESC colonies were excluded from the analysis. The average size of the HS237 and HS293 colonies at day 6 was 0.99 mm^2 ^(± 0.61, *n *= 24), with 91% undifferentiated cells and 9% differentiated cells, according to the automated analysis protocol.

To confirm the results of the automated analysis, some of the hESC colonies were also analyzed by FACS, and the rest were stained immunohistochemically. Analysis by FACS of the pooled HS237 colonies (*n *= 143), with a total of 3.0 million cells at day 7, revealed that an average size colony of 1.04 mm^2 ^contained ~21000 cells (n = 29). The analysis (in triplicate) revealed that 81% of the HS237 cells were positive for SSEA4 (Figure 2d). This result was consistent with the automated area protocol result, which indicated that 86.9% of the HS237 colony area (*n *= 29) was undifferentiated. Immunohistochemical staining of the automatically monitored colonies showed that these colonies were positive for Nanog and that SSEA1 was expressed in only a small proportion (3%) of the cells (Figure 2f). Both Nanog and SSEA-4 are widely considered as markers for undifferentiated hESCs although they may not always monitor the same cell population. SSEA-1 was used as common marker for differentiated cells since the aim was not to address towards which lineages the cells were differentiating.

**Figure 3:**
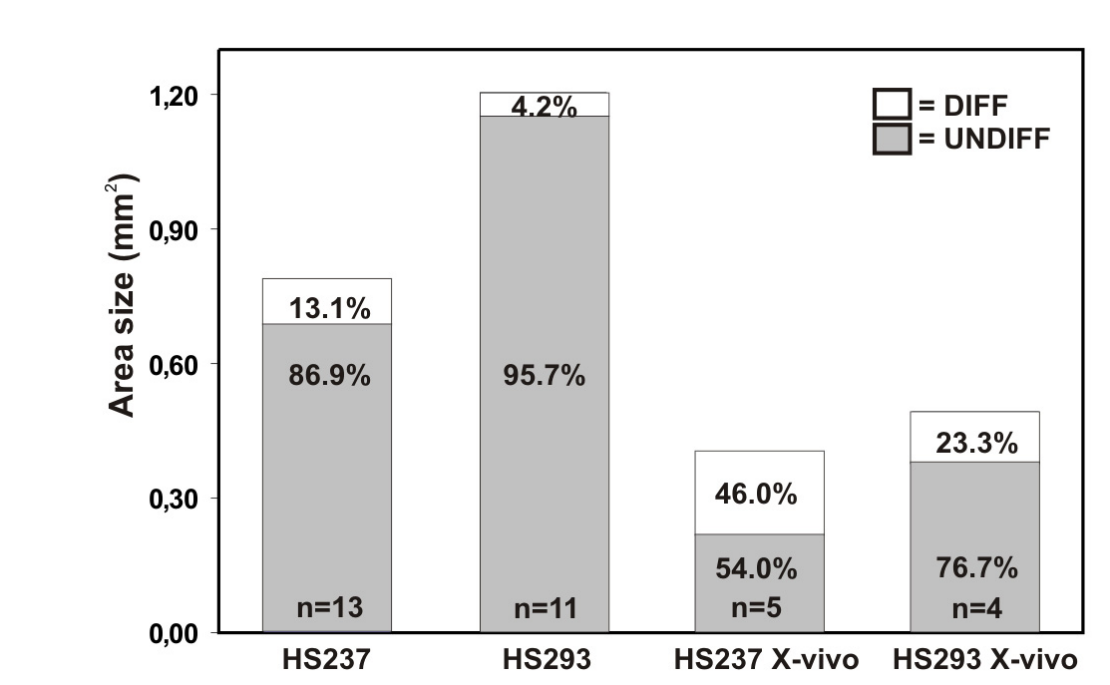
Results of an automated area analysis of HS237 and HS293 hESC lines. A bar chart representing the proportions of undifferentiated and differentiated areas of hESC colonies cultured in conventional hESC medium and in X-vivo 10 medium.

Parallel cell culture plates were cultured in conventional incubators in otherwise similar conditions. The colonies grown in parallel plates were observed by an experienced observer similarly with routine every day evaluation practise. There were no differences in the growth rates or in the areas of undifferentiated and differentiated cells between the hESC colonies cultured in a standard incubator or in the automated system based on the colony size. Typically, spontaneous differentiation of undifferentiated colonies started six to eight days after passaging in the center of the colonies (data not shown). This phenomenon occurred similarly in the colonies grown in the Cell-IQ system and in a common incubator. Also, there were similar portion of totally differentiated and non-growing hESC colonies in the plates that were grown in Cell-IQ system and in a common incubator. As FACS and immunohistochemical analyses confirmed that the automated analysis protocol recognized undifferentiated and differentiated areas reliably, we further examined the growth parameters of HS237 and HS293 lines. There was no difference in the average colony area between lines HS237 (0.79 ± 0.32 mm^2^) and HS293 (1.20 ± 0.80 mm^2^) at day 6 after passaging. The growth rates of the undifferentiated areas (from days 5 to 6) of HS237 (215 ± 94 μm^2^/min) and HS293 (328 ± 187 μm^2^/min) were also similar. There were more undifferentiated areas in HS293 colonies than in HS237 colonies (*P *< 0.01, Figure 3).

The development of animal component-free culture conditions for hESCs is a major challenge [12]. In this study, we tested whether the automated culturing and monitoring system could be used for the testing of culture media. Two different media were used: conventional hESC culture medium and an X-vivo 10-based, xeno-free culture medium. Both HS237 and HS293 colonies grew better in conventional medium than in X-vivo 10 medium [see Additional file 5]. The average colony size (both HS237 and HS293) in the X-vivo 10 medium was smaller than in conventional hESC medium (0.44 ± 0.31 vs. 0.99 mm^2 ^± 0.61, *P *< 0.01, Figure 3), and the undifferentiated cell area was smaller in colonies cultured in the X-vivo 10 medium (64%) than in conventional hESC medium (91%, *P *< 0.01).

The continuous monitoring of living cells revealed more information than conventional microscopic observation of cultures. With the aid of the automated system we were able to observe cell behavior that would be impossible to discover by conventional microscopic observation. Traditionally, the growth rates of hESC colonies have been manually counted from single cell suspensions as doubling time during the exponential growth phase [13] or as an expansion of the area of a single colony from microscopic images [14]. The automated monitoring system allowed a reliable area measurement of live, unlabeled, intact cell colonies. Hence, we obtained continuous quantitative data from the cultures with constant real-time feedback. In culture medium testing, the automated machine vision culture platform, revealed that both hESC lines grew better in conventional hESC medium than in X-vivo 10 medium.

## Conclusion

The automated cell culturing and analysis system provides an optimal tool for the evaluation of hESC cultures, allowing continuous well-to-well comparison of the effects of different culture media or different growth factor concentrations on cell growth and behaviour – such as differentiation. In addition, it can also be used as a tool in the optimization of differentiation protocols for hESCs. Whether this system can be applied to monitoring and analysis of 3D cell differentiation remains to be studied. Our data clearly demonstrate that continuous monitoring of living cell cultures without disturbing the cells can reveal more information than conventional microscopic observation of cultures. The automated system enables rapid and reliable analysis of undifferentiated growth dynamics of hESCs.

## Competing interests

The author(s) declare that they have no competing interests.

## Authors' contributions

S.N. performed the Cell-IQ experiments and data analyses and contributed to writing the manuscript. K.R. performed the cell culture and characterization experiments and contributed to writing the manuscript. H.P. and R.S. contributed to the conception and design of the experiments. O.H. contributed to the conception, and design of the experiments, interpretation of data, and critically revising the manuscript for important intellectual content. H.S. contributed to the conception, and design of the experiments, interpretation of data, and to writing the manuscript.

## Supplementary Material

Additional File 1 Representative sample images of user-defined classes for morphologic analysis of hESC colonies. For the analysis **(a) **background feeder cells, **(b) **undifferentiated, and **(c) **differentiated hESC areas were defined as separate classes. One sample image presents an area of 56.3 × 56.3 μm as shown in **(a) **by the dotted square. Adequate numbers of sample images captured by the system were compared with visual evaluation of the colonies using a conventional microscope to ensure that correct demonstrative areas were chosen for the area analysis protocol of the automated system. Click here for file

Additional File 2 The diagram representing two phases required for the analysis. The classifier must be created once (the topmost diagram) using the set of cell images, and then it can be used (the lower diagram) for the analysis of the captured images. Click here for file

Additional File 3 The resulting image of an hESC colony cultured in a conventional hESC medium. (a) The hESC colony was analyzed using the defined area analysis protocol. The resulting image of the analyzed colony is shown in (b). Green color represents undifferentiated areas and blue represents differentiated areas according to the analysis result. (c) The hESC colony was immunohistochemically characterized using an antibody against an undifferentiated hESC marker, Nanog (green), and a differentiated hESC marker, SSEA1 (red). Scale bar = 200 μm. Click here for file

Additional File 4 The growth of an hESC colony in conventional hESC medium. The growth of an hESC colony from day 3 until day 7 in conventional hESC medium. Click here for file

Additional File 5 The growth of an hESC colony in X-vivo 10 medium. The growth of an hESC colony from day 3 until day 7 in X-vivo 10 medium. Click here for file
