# Supplementary figures and images for: Monitoring and analysis of dynamic growth of human embryonic stem cells: comparison of automated instrumentation and conventional culturing methods
Source: Biomed Eng Online. 2007 Apr 12;6:11. doi: 10.1186/1475-925X-6-11 (PMC1854905; doi:10.1186/1475-925X-6-11)

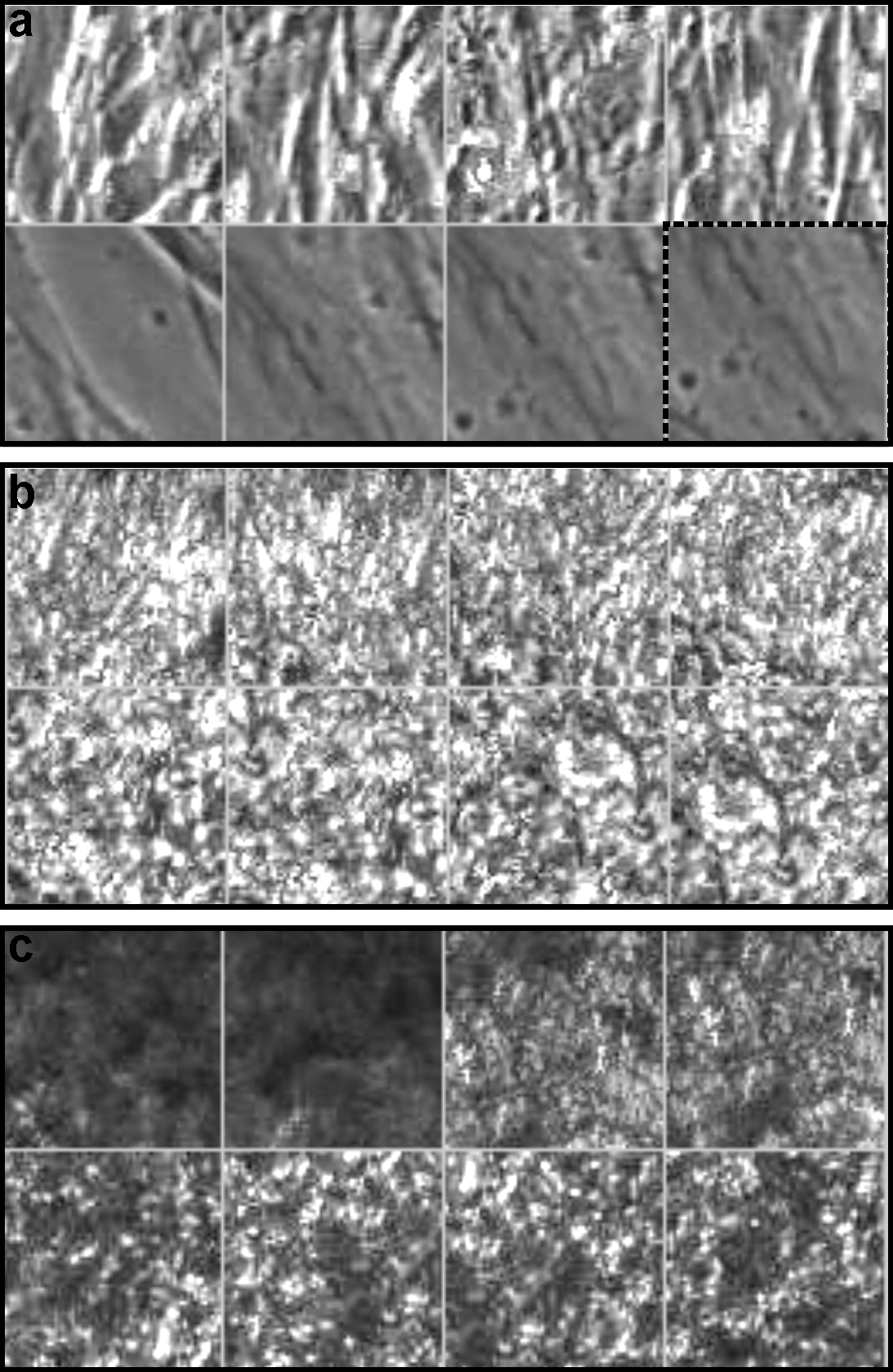

Supplement: Additional File 1 — Representative sample images of user-defined classes for morphologic analysis of hESC colonies. For the analysis (a) background feeder cells, (b) undifferentiated, and (c) differentiated hESC areas were defined as separate classes. One sample image presents an area of 56.3 × 56.3 μm as shown in (a) by the dotted square. Adequate numbers of sample images captured by the system were compared with visual evaluation of the colonies using a conventional microscope to ensure that correct demonstrative areas were chosen for the area analysis protocol of the automated system. [file 1475-925X-6-11-S1.tiff]

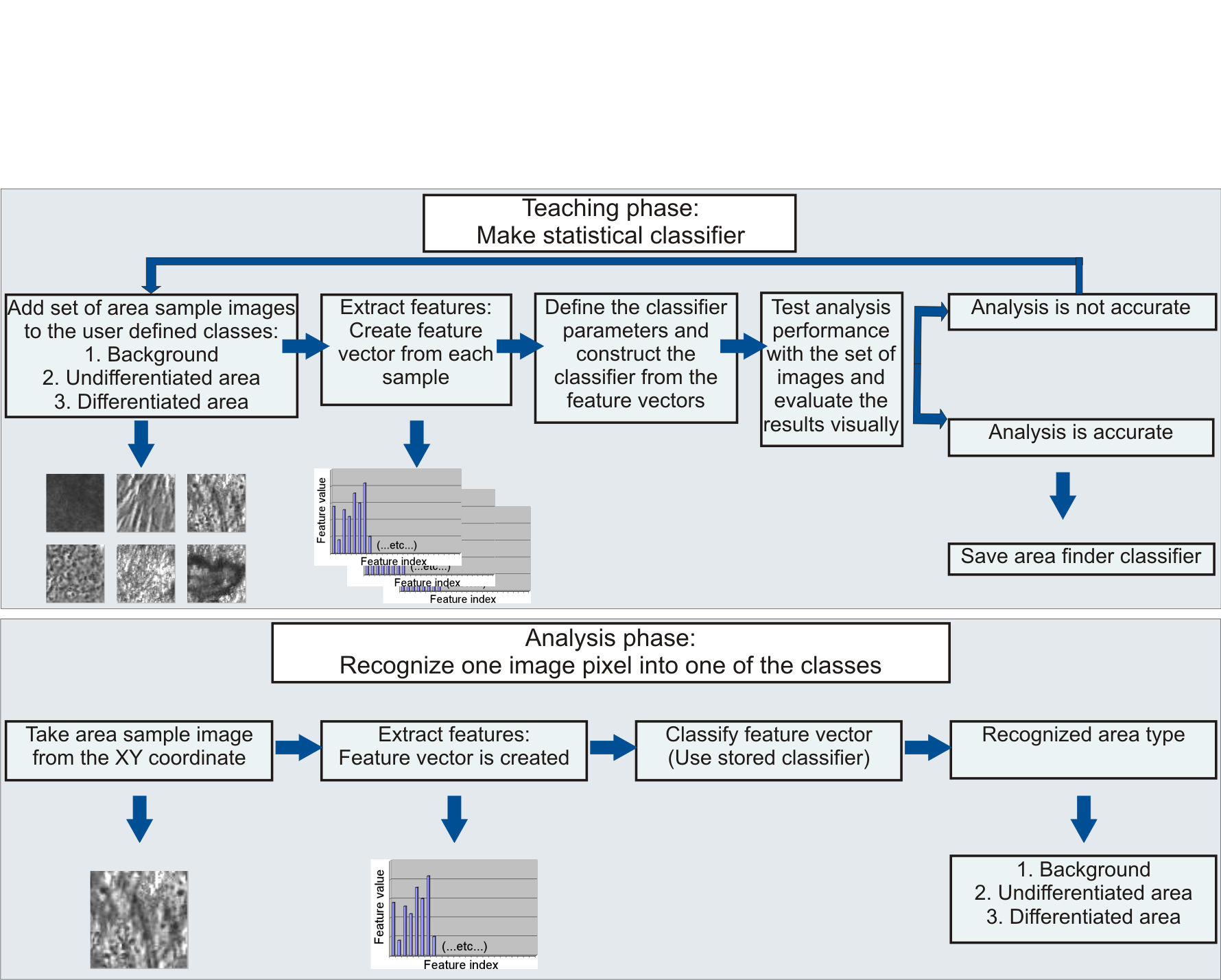

Supplement: Additional File 2 — The diagram representing two phases required for the analysis. The classifier must be created once (the topmost diagram) using the set of cell images, and then it can be used (the lower diagram) for the analysis of the captured images. [file 1475-925X-6-11-S2.tiff]
